# Synthetic polyploidization induces enhanced phytochemical profile and biological activities in *Thymus vulgaris* L. essential oil

**DOI:** 10.1038/s41598-024-56378-7

**Published:** 2024-03-07

**Authors:** Neha Gupta, Soham Bhattacharya, Adrish Dutta, Jan Tauchen, Přemysl Landa, Klára Urbanová, Markéta Houdková, Eloy Fernández-Cusimamani, Olga Leuner

**Affiliations:** 1 Department of Crop Sciences and Agroforestry, Faculty of Tropical AgriSciences, Czech University of Life Sciences Prague, Kamýcká 129, 165 00 Suchdol, Prague 6, Czech Republic; 2 Department of Agroecology and Crop Production, Faculty of Agrobiology, Food and Natural Resources, Czech University of Life Sciences Prague, Kamýcká 129, Suchdol, Prague 6, 165 00 Czech Republic; 3 Department of Food Science, Faculty of Agrobiology, Food and Natural Resources, Czech University of Life Sciences, Prague, Czech Republic; 4 Laboratory of Plant Biotechnologies, Institute of Experimental Botany of the Czech Academy of Sciences, Rozvojova 263, 165 02 Lysolaje, Prague 6, Czech Republic; 5 Department of Sustainable Technologies, Faculty of Tropical AgriSciences, Czech University of Life Sciences Prague, Prague, Czech Republic

**Keywords:** Biochemistry, Biological techniques, Biotechnology, Microbiology, Molecular biology, Plant sciences

## Abstract

Essential oil from *Thymus vulgaris* L. has valuable therapeutic potential that is highly desired in pharmaceutical, food, and cosmetic industries. Considering these advantages and the rising market demand, induced polyploids were obtained using oryzalin to enhance essential oil yield. However, their therapeutic values were unexplored. So, this study aims to assess the phytochemical content, and antimicrobial, antioxidant, and anti-inflammatory activities of tetraploid and diploid thyme essential oils. Induced tetraploids had 41.11% higher essential oil yield with enhanced thymol and γ-terpinene content than diploid. Tetraploids exhibited higher antibacterial activity against all tested microorganisms. Similarly, in DPPH radical scavenging assay tetraploid essential oil was more potent with half-maximal inhibitory doses (IC_50_) of 180.03 µg/mL (40.05 µg TE/mg) than diploid with IC_50_ > 512 µg/mL (12.68 µg TE/mg). Tetraploids exhibited more effective inhibition of in vitro catalytic activity of pro-inflammatory enzyme cyclooxygenase-2 (COX-2) than diploids at 50 µg/mL concentration. Furthermore, molecular docking revealed higher binding affinity of thymol and γ-terpinene towards tested protein receptors, which explained enhanced bioactivity of tetraploid essential oil. In conclusion, these results suggest that synthetic polyploidization using oryzalin could effectively enhance the quality and quantity of secondary metabolites and can develop more efficient essential oil-based commercial products using this induced genotype.

## Introduction

*Thymus vulgaris* L., popularly known as garden thyme or common thyme, is a perennial bushy and wood-based aromatic herb belonging to the Lamiaceae family predominantly found in the Mediterranean regions, Asia, Southern Europe, and North Africa^1^. The genus *Thymus* comprises approximately 400 species, widely used in traditional for treating ailments such as cough, bronchitis, sore throat, arthritis, and rheumatism^2–5^ in addition to its culinary use for taste enhancement and preventing food spoilage^6^. Thymol is found in abundance in *T. vulgaris* followed by carvacrol, geraniol, α-terpineol, 4-thujanol, linalool, 1,8-cineole, myrcene, γ-terpinene, and *p*-cymene solely responsible for antitussive and antibroncholitic^7^, antispasmodic anti-cancer, and several medicinal properties^8–10^. The bioactivities of *T. vulgaris* essential oil mostly depend on its terpene and terpenoid contents^11,12^. There has been a plethora of reports validating the in vitro antibacterial activity of thyme essential oil on some respiratory disease-causing pathogens including *Staphylococcus aureus, Pseudomonas aeruginosa, Haemophilus influenza,* and *Streptococcus pneumonia*^13,14^. Thyme essential oil is used commercially as a natural preservative in food industries to prevent spoilage as well as for food packaging systems^15–17^.

Essential oil-based products promote organic, natural, and green consumerism throughout the global marketplace leading to escalating rivalry to create high-quality cultivars and to reduce production costs. The gap between the supplier and the market demand created due to the lesser yield of essential oils and heavy use of labor has led to over-harvesting from the wild causing gene-pool deficiency^18^. Several pieces of literature highlighted the fact that geographical, environmental, agroclimatic, and various genetic factors can influence the quantity of essential oil production in the plant along with chemical composition and its biological activity^19–21^.

Keeping in mind that consumers prefer natural products over genetically modified plants due to safety issues^22^ and other conventional plant breeding techniques are quite expensive and time-consuming, synthetic polyploidization is considered one of the safest and ideal contemporary breeding approaches^23,24^. Synthetic polyploidization is chromosome duplication of the whole genomic constitution of an organism creating genetic uniqueness using chemical antimitotic agents like oryzalin, colchicine, trifluralin, etc.^25^, may result in superior or inferior genotypes with enhanced or reduced morphological, physiological, and biochemical properties as the result of gene duplication is unknown^26^. It is reported that oryzalin has fewer side effects compared to colchicine and possesses a higher affinity towards plant tubulin^27^.

Several studies reported and it is often hypothesized that synthetic polyploidization enhanced primary and secondary metabolite production due to chromosome doubling that influenced the biological activities of the polyploid plants^28–32^. However, one of such reports has proven it as a myth^33^. Thus, it is important to assess the quality of these induced genotypes. However, the bio-activity analysis of polyploid plants’ secondary metabolites is still in its budding stage, and as of now, there has been no research article reporting the biological activities of induced tetraploid *T. vulgaris* essential oil by broadening the concern on antimicrobial, anti-inflammatory, and antioxidant activities. Therefore, the objective of this study was to extract essential oil from induced tetraploid thyme plants and characterize the effectiveness of polyploidization on *T. vulgaris* essential oil on its chemical composition and biological activities such as antimicrobial, anti-inflammatory, and antioxidant activities compared to essential oil extracted from diploid thyme. The current findings may elucidate the increase of secondary metabolite production in the polyploid genotype through synthetic polyploidization that positively influences the biological activities of plants that are of great economic importance to the pharmaceutical, cosmetic, and food industries.

## Materials and methods

### Plant material acquisition

Artificially induced autopolyploid plants of *T. vulgaris* (2n = 4x = 60) and *T. vulgaris* control plants (2n = 2x = 30) were obtained from the previous study by Homaidan Shmeit et al., 2020^34^ and maintained in the field condition at the botanical garden of the Faculty of Tropical Agrisciences (FTA), Czech University of Life Sciences Prague (CZU). The control *T. vulgaris* plants were obtained from the botanical garden (Index seminum number-343, year: 2019) identified by Marie Hlaváčová (Botanist and Curator) of botanical garden FTA, CZU. The plant materials were not deposited in the herbarium repository as they were obtained from seeds and through plant tissue culture for experimental purposes and later maintained in the field of the botanical garden. For experimental purposes, the plant materials were collected from the parental plants maintained in field conditions at the botanical garden of FTA, CZU (50.131115 N, 14.370528 E) with permission and relevant institutional guidelines. The flow cytometric analysis was conducted as a confirmatory test using a Partec PAS flow cytometer (Partec GmbH, Munster Germany) equipped with a high-pressure mercury arc as described by Bharati et al.^35^ and the results can be found in Supplementary (Fig. S1a-b).

### Essential oil extraction

Fresh aerial parts of *T. vulgaris* were obtained from tetraploid and diploid control plants and dried at 30 °C. Dried samples were then ground and homogenized using a Grindomix apparatus (GM 100 Retsch, Haan, Germany). The residual moisture content was evaluated gravimetrically in triplicate by Scaltec SMO 01 Analyzer (Scaltec Instruments, Gottingen, Germany) at 130 °C for 1 h and expressed as arithmetic averages. Ground samples were then hydro-distilled using a Clevenger-type apparatus. The extracted essential oils were collected in air-tight glass vials and stored at 4 °C until further use.

### Chemical analysis of essential oils

Chemical characterization of essential oils has been done using the Agilent GC-7890B system (Agilent Technologies, Santa Clara, CA, USA) equipped with autosampler Agilent 7693, non-polar HP-5MS column (30 m × 0.25 mm, film thickness 0.25 μm, Agilent 19091 s-433), and a flame ionization detector (FID) coupled with single quadrupole mass selective detector Agilent MSD-5977B. Samples were diluted in n-hexane for GC–MS analysis at a concentration of 20 μl/mL. 1 μl of the solution was injected in splitless mode. The injector temperature was 250 °C. The initial temperature of the oven was 60 °C for 1 min and then increased to 240 °C at a rate of 3 °C/min. The transfer line temperature was kept at 250 °C. We used helium as a carrier gas and the flow rate was 1 ml/min. The FID was programmed with a heating temperature of 250 °C, an H_2_ flow rate of 40 ml/min, an airflow rate of 400 ml/min, and a make-up flow rate of 30 ml/min. The MS analysis was carried out with the following conditions: ionization energy 70 eV, ion source temperature 230 °C, and mass range 30–550 m/z. The identification of chemical components was based on the comparison of their retention indices (RIs), retention times (RT), spectra with the National Institute of Standards and Technology Library (NIST 2.0.f), and the available literature^36^. The RI of the separated compounds was calculated using the retention times of the n-alkanes series ranging from C8 to C40 (Sigma-Aldrich, Prague, Czech Republic). The relative percentage content of chemical components was determined from FID.

### Bacterial strain and culture media

For the antimicrobial assay, American Type Culture Collection (ATCC) that includes *Haemophilus influenzae* ATCC 49247, *Staphylococcus aureus* ATCC 29213, *Streptococcus pneumoniae* ATCC 49619, and *Streptococcus pyogenes* ATCC 19615 was used. The cultivation and assay media (broth/agar) were Mueller Hinton (MH), complemented with Haemophilus Test Medium and defibrinated horse blood for *H. influenzae*, MH only for *S. aureus,* and Brain Heart Infusion for both *S. pneumoniae* and *S. pyogenes*. The pH of the broth was adjusted to a final value of 7.6 using Trizma base (Sigma-Aldrich, Prague, Czech Republic). All microbial strains, growth media, and other supplements were purchased from Oxoid (Basingstoke, Hampshire, UK).

Stock cultures of bacterial strains were cultivated in appropriate media at 37 °C for 24 h before testing. The turbidity of the bacterial strains was adjusted to 0.5 McFarland standard using Densi-La-Meter II (Lachema, Brno, Czech Republic) to reach the final concentration of 10^7^ CFU/mL. Ampicillin and amoxicillin were purchased from Sigma-Aldrich (Prague, Czech Republic) and assayed as positive antibiotic controls for all the strains used (CLSI).

### Antimicrobial assay

In vitro growth-inhibitory effect of essential oils was assessed using the Broth Microdilution Volatilization (BMV) method that allows the assessment of the antibacterial activity of essential oils at different concentrations in both liquid and vapor phases as described by Hudokova et al.^37,38^. A standard 96-well microtiter plate (well volume = 400 µL) with tight-fitting lids and flanges was used for this experiment. Each essential oil sample was dissolved in dimethyl sulfoxide (DMSO) (Sigma-Aldrich, Prague, Czech Republic) at a maximum concentration of 1% and diluted in appropriate broth medium, and seven two-fold serial dilutions of samples of all essential oils starting from 1024 µg/mL were prepared with 100 µL as the final volume of each well. The plates were then inoculated with bacterial suspension using a 96-pin multi-blot replicator (National Institute of Public Health, Prague, Czech Republic) and incubated at 37 °C for 24 h. The wells containing inoculated and non-inoculated broth were simultaneously prepared for growth and purity controls. The Minimum Inhibitory Concentrations (MIC) were evaluated by visual assessment of bacterial growth after the coloring of metabolically active bacterial colonies with thiazolyl blue tetrazolium bromide dye (MTT) at a concentration of 600 µg/mL (Sigma-Aldrich, Prague, Czech Republic). The MIC values were determined as the lowest concentrations that inhibited the bacterial growth compared to compound-free control and expressed in µg/mL (1024, 512, 256, 128, 64, 32, and 16 µg/mL, respectively). In the case of vapor phase, these concentrations can be expressed as weight of volatile agent per unit volume of a well and the MIC values would be expressed in µg/cm^3^ (256, 128, 64, 32, 16, 8, and 4 µg/cm^3^, respectively). All the experiments were performed in triplicate with three independent measurements and the results were expressed as median/modal MIC values.

### Antioxidant activity

The radical scavenging assay using DPPH (2,2-diphenyl-1-picrylhydrazyl) was tested to determine the ability of samples to scavenge the DPPH radicals using the method described by Stastny et al.,^39^. Samples were diluted in analytical-grade methanol to obtain the initial concentration of 1024 μg/mL. Subsequently, the serial dilution of each sample was prepared in methanol (100 μL) in 96-well microtiter plates. The radical scavenging reaction was started after the addition of 100 μL of freshly prepared 0.25 mM DPPH in methanol to each well along with samples, thus creating a range of 512 to 0.5 μg/mL. Trolox was used as a standard reference material and pure methanol as a blank control. The absorbance was measured at 517 nm using Synergy H1 multi-mode reader (BioTek, Winooski, Winooski, VT, USA). The results were expressed as half-maximal inhibitory concentrations (IC_50_ in μg/mL) and Trolox equivalents (mg TE/g extract).

### In vitro* anti-inflammatory activity*

For evaluating anti-inflammatory activity, the inhibitory activity against cyclooxygenases was determined using the previously described method by Langhansova et al.,^40^ with slight modifications. COX-2 (0.125 units/reaction) was added to 180 µL of incubation mixture consisting of 100 mM Tris buffer (pH 8.0), 5 µM hematin porcine, 50 µM Na_2_EDTA, and 18 mM L-epinephrine. The essential oil samples were dissolved in DMSO and 10 μL was added to incubation mixture in the 96-well microplate with 5 μL of COX enzyme. After adding 10 µM arachidonic acid the reaction was initiated. After 20 min of incubation at 37 °C, the reaction was ceased by adding 10 μL of 10% formic acid. The PGE_2_ concentration was determined by PGE_2_ ELISA kit according to the manufacturer’s instructions and the final solutions were diluted at 1:15 in assay buffer. The absorbance was measured with a microplate reader (Tecan Infinite M200) at 405 nm and the inhibitory activity was calculated as the percentage inhibition of PGE_2_ production compared to blank. (S)-(+)-ibuprofen was used as a reference inhibitor and DMSO as the blank. The experiment was repeated at least two times with at least two technical replicates in each experiment.

### Molecular docking study

Molecular docking was done to understand the interaction of major compounds identified from essential oils with bacterial protein receptors along with confirming their antioxidant and anti-inflammatory properties. The docking study was conducted according to the previously described method by Gupta et al.,^41^. The crystal structure of seven universal bacterial proteins such as isoleucyl-tRNA synthetase (PDB ID: 1JZQ), DNA gyrase (PDB ID: 1KZN), dihydropteroate synthase (PDB ID: 2VEG), D-alanine: D-alanine ligase (PDB ID: 2ZDQ), topoisomerase IV (PDB ID: 3RAE), dihydrofolate reductase (PDB ID: 3SRW), penicillin-binding protein 1a (PDB ID: 3UDI), and also protein human cyclin-dependent kinase 2 complex (PDB ID: 1HCK), and cyclooxygenase-2 (PDB: 1CX2) were obtained from Protein Data Bank (https://www.rcsb.org/, accessed on 12 November 2023). Ascorbic acid was used in antioxidant activity as a positive reference as described by Mendes-da-Silva et al.,^42^. Each center and size were submitted to AutoDock Tools (https://autodock.scripps.edu/download-autodock4/) for docking using the interface of the command prompt and the interaction and visualization were performed for the best-docked complexes using LigPlot + ver. 2.2 (https://www.ebi.ac.uk/thornton-srv/software/LigPlus/download.html).

### Statistical analysis

The data obtained from the antioxidant and anti-inflammatory activity of control diploid and induced polyploid genotypes were presented as means ± SD. The IC_50_ of antioxidant activity (half-maximal inhibitory concentration) was calculated by plotting the values for % inhibition (absorbance_blank_ − absorbance_sample_/absorbance_blank_ × 100) to the particular concentration of the sample/positive control. The IC_50_ was expressed as the concentration (in μg/mL) corresponding to the 50% inhibition of the DPPH radical with the use of the Gen5 microplate and imager software ver 3.04 (BioTek, Winooski, USA) (https://www.agilent.com/en/product/cell-analysis/cell-imaging-microscopy/cell-imaging-microscopy-software/biotek-gen5-software-for-imaging-microscopy-1623226). All obtained values from essential oil yield, antioxidant, and anti-inflammatory activity were analyzed and compared based on Tukey's post hoc analysis (5% significance level) in the Microsoft Excel 2021 software package (https://softwarekeep.eu/microsoft-office-2021-home-and-student-pc.html).

### Ethical statement

All experiments conducted in this study, including essential oil extraction (according to European Pharmacopoeia)^43^, antimicrobial activity (according to CLSI)^44^, and the collection of plant material, comply with relevant institutional, national, and international guidelines and legislation.

## Results

### Essential oil yield and chemical composition

**Figure 1:**
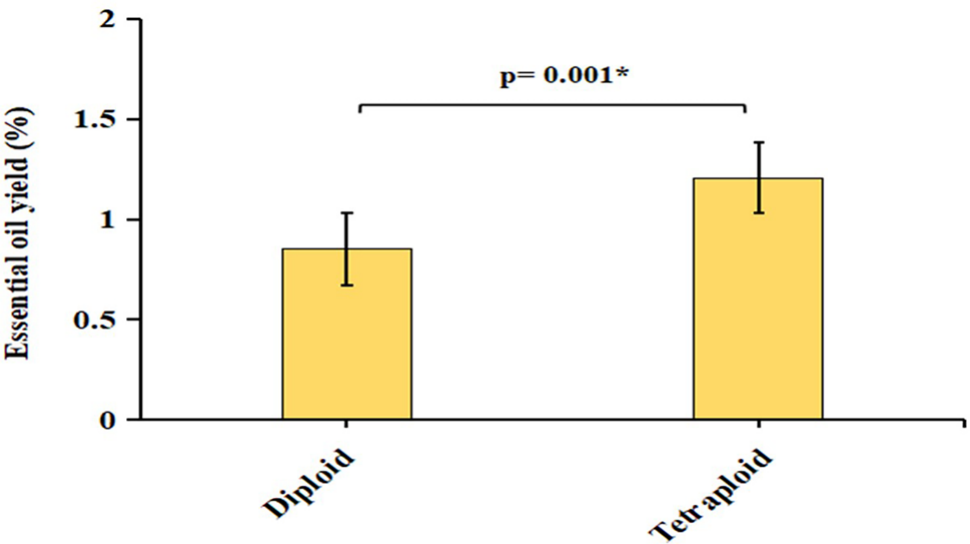
Essential oil yield in control diploid and induced polyploid of T. vulgaris. “*” expressed a significant difference (Tukey HSD Test, p < 0.05).

*T. vulgaris* essential oils from tetraploid and diploid genotypes were extracted by hydro-distillation method with an average moisture content of 12.77% and 13.45%, respectively. The essential oil yield values were 1.2% and 0.85% in tetraploid and diploid plants, respectively. The polyploid plants yielded higher amount of essential oil than the control diploid (Fig. 1). All essential oils presented a strong fragrance with a palish-yellow color. GC–MS analysis resulted in the identification of 16 compounds in total in both genotypes representing 99.86% (diploid) and 99.88% (polyploid) of their corresponding total constituents (Table 1) as well as Supplementary (Fig. S2a,b). Monoterpenoid represented by thymol was the most abundant compound found in tetraploid and diploid (53.5% and 50.65%, respectively), followed by monoterpenes comprising of γ-terpinene and *p*-cymene constituting 21.81% and 7.85% in tetraploid and 5.55% and 20.40% in diploid, respectively. The major compounds considered were based on the significant amount of the total composition present in both diploid and polyploid essential oils which is above 5%. These three major compounds together constituted 83.16% and 76.67% of the total composition of tetraploid and diploid *T. vulgaris* essential oil, respectively. To a lesser extent, other compounds were identified such as caryophyllene (5.8% and 2.13%), caryophyllene oxide (0.67% and 1.71%), borneol (1.34% and 3.78%), d-camphor (0.21% and 4.21%), eucalyptol (1.01% and 3.74%) which had significant differences between tetraploid and diploid genotypes, respectively. The differences in the content of other components did not exceed 1%.

**Table 1:** Essential oil constituents of diploid (Control) and polyploid genotype of *T. vulgaris*.

Compound name	RI^a^		Content [%]^c^	
	Observed	Literature^b^	Diploid	Tetraploid
3-Thujene	926	931	0.26	0.30
α-Pinene	933	937	0.54	0.24
Camphene	948	953	0.87	0.16
Myrcene	991	991	0.68	0.76
4-Carene	1017	1009	0.70	1.5
**p-Cymene**	1026	1026	**20.40**	**7.85**
Eucalyptol	1031	1033	3.74	1.01
**γ-Terpinene**	1060	1062	**5.55**	**21.81**
Linalool	1103	1098	2.82	2.79
d-Camphor	1146	1143	4.21	0.21
Borneol	1171	1165	3.78	1.34
4-Terpineneol	1181	1175	1.70	1.29
**Thymol**	1307	1290	**50.65**	**53.5**
Caryophyllene	1423	1418	2.13	5.8
D-Germacrene	1485	1480	0.12	0.65
Caryophyllene oxide	1589	1581	1.71	0.67
**Total**			**99.86**	**99.88**

a: Kovats’ retention indices measured on HP-5MS column; b: retention indices from literature; c: relative percentage content based on the total area of all peaks^45^.

Major compounds are in [bold].

### Antimicrobial activity

Samples of essential oil from diploid and tetraploid *T. vulgaris* were tested against four standard bacterial strains related to respiratory infections (Table 2). All essential oils offered a certain degree of antibacterial efficacy ranging from 128 to 1024 μg/mL in both liquid and vapor phases. The tetraploid essential oil was the most active with the lowest MIC value of 128 μg/mL in liquid and 1024 μg/mL in vapor phase whereas, diploid essential oil showed the lowest MIC value of 256 μg/mL in liquid and 1024 μg/mL in vapor phase. *H. influenzae* growth was most sensitive to *T. vulgaris* essential oil where tetraploid presented a MIC value of 128 μg/mL and diploid presented a MIC value of 256 μg/mL in the liquid phase. However, in the vapor phase both essential oils presented a MIC value of more than 1024 μg/mL. Mild activity against *S. pyogenes* and *S. aureus* (512 μg/mL) for tetraploid and weak activity (1024 μg/mL) for diploid in the liquid phase was recorded. For vapor phase, MIC value of 1024 μg/mL was observed for both essential oils against *S. pyogenes,* however, a concentration greater than 1024 μg/mL was needed for diploid essential oil against *S. aureus.* Against *S. pneumoniae* both essential oils showed the same activity (512 μg/mL and 1024 μg/mL) for liquid and vapor phases, respectively. It was observed that both essential oils were more effective in the liquid phase against all tested microorganisms than in the vapor phase.

**Table 2:** In vitro growth-inhibitory effect of *T. vulgaris* L. essential oils (control and tetraploid) in liquid and vapor phases against respiratory infection bacteria.

Bacterium/growth medium/minimum inhibitory concentration								
Essential oil	*Haemophilus influenzae*		*Staphylococcus aureus*		*Streptococcus pneumoniae*		*Streptococcus pyogenes*	
	Agar*	Broth	Agar	Broth	Agar	Broth	Agar	Broth
	(µg/mL)	(µg/mL)	(µg/mL)	(µg/mL)	(µg/mL)	(µg/mL)	(µg/mL)	(µg/mL)
Diploid	> 1024	256	> 1024	1024	1024	512	1024	1024
Tetraploid	> 1024	128	1024	512	1024	512	1024	512
Positive antibiotic control								
Amoxicillin	NT	NT	NT	NT	> 2	> 2	NT	NT
Ampicillin	> 2	0.25	> 2	2	NT	NT	> 2	2

NT: Not tested; *: If the distribution of volatiles is uniform in liquid and gaseous phase, the concentrations can be expressed as weight of volatile agent per volume unit of a well, whereas their real values will be 256, 128, 64, 32, 16, 8, 4 and 2 μg/cm^3^ for 1024, 512, 256, 128, 64, 32, 16, and 8 μg/mL, respectively.

### Antioxidant activity

The DPPH radical scavenging assay was used for the screening of the antioxidant activity between *T. vulgaris* diploid and polyploid essential oils. The antioxidant activity results are summarized as IC_50_ and TE (Trolox equivalent) in Table 3. It was observed from the results that the tetraploid essential oil was more potent in inhibiting the DPPH radical. The antioxidant activity of essential oil from tetraploid thyme was stronger with half-maximal inhibitory concentrations (IC_50_) of 180.03 ± 51.50 µg/mL (40.05 ± 14.01 µg TE/mg), while the diploid essential oil was found to be an IC_50_ value of more than 512 µg/mL (less than 12.68 µg TE/mg). The antioxidant activities were found highly significant (*p* < 0.05) in the tetraploid compared to the diploid. It was also observed that none of the tested essential oils had significantly higher activity as compared to trolox (IC_50_ 6.49 ± 1.01 µg/mL). The tetraploid genotype possessed the highest DPPH radical scavenging activity and therefore, has the ability to prevent oxidative stress better than the diploid genotype.

**Table 3:** Antioxidant activity of *T. vulgaris* diploid and polyploid essential oils.

Plant sample	DPPH	
	IC_50_ ± SD^1^(µg/mL)	µg TE/mg ± SD
Diploid	> 512	> 12.68
Tetraploid	180.03 ± 51.50*	40.05 ± 14.01*
Positive control Trolox	6.49 ± 1.01	-

^1^IC_50_ ± SD: half maximal inhibitory concentration ± standard deviation; TE = Trolox equivalent; *Shows a significant difference between the diploid and tetraploid genotype based on Tukey's test for post hoc analysis at 5% significance level.

### Anti-inflammatory activity

In vitro anti-inflammatory activity of *T. vulgaris* diploid and polyploid essential oils was tested as inhibition of COX-2 catalytic activity. In comparison to untreated control, the PGE_2_ production was significantly reduced in the presence of 500 and 50 µg/mL of essential oils whereas they were inactive at 5 µg/mL concentration. The activity of both essential oils was comparable (Table 4). The anti-inflammatory activity between tetraploid and diploid essential oil was found to be significant (*p* < 0.05) at 50 µg/mL concentration with an inhibition value of 83.74 ± 5.8 and 70.53 ± 11.86 respectively.

**Table 4:** In vitro anti-inflammatory activity of *T. vulgaris* diploid and polyploid essential oils determined by inhibition of COX-2 enzyme.

Samples	Concentration (µg/mL)	Inhibition %
Diploid	500	80.96 ± 7.2
	50	70.53 ± 11.86
	5	2.02 ± 17.13
Tetraploid	500	85.57 ± 7.5
	50	83.74 ± 5.8*
	5	6.74 ± 23.48
Ibuprofen	5	74.52 ± 10.2

The results are expressed as means ± SD for two independent experiments measured in duplicate. The results were compared by Tukey's test for post hoc analysis at 5% significance level. *Shows a significant difference between the diploid and tetraploid genotypes.

### Molecular docking

**Figure 2:**
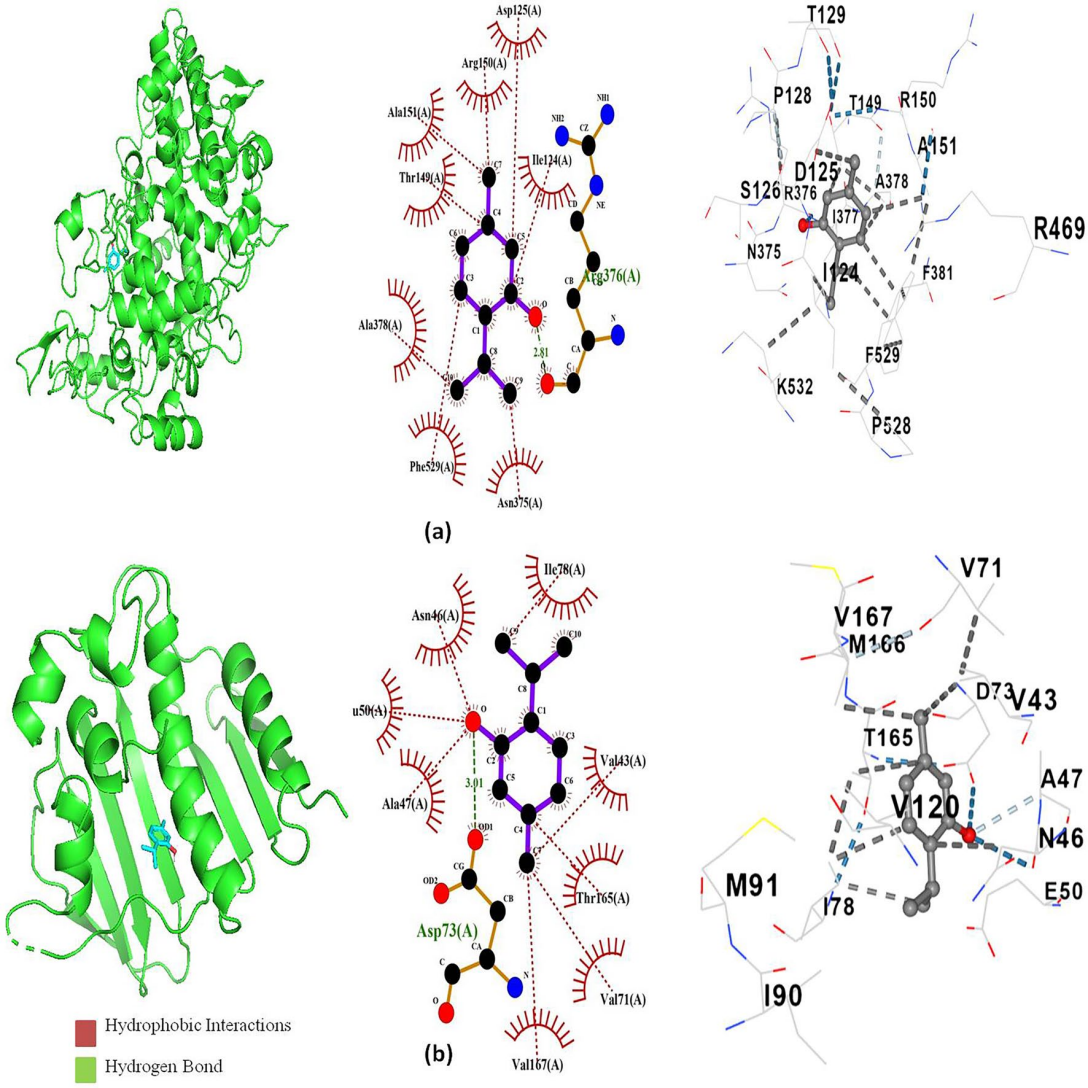
Interactions and docked 3D structures of (**a**) thymol with cyclooxygenase-2 enzyme 1CX2, (**b**) thymol with DNA gyrase 1KZN.

**Figure 3:**
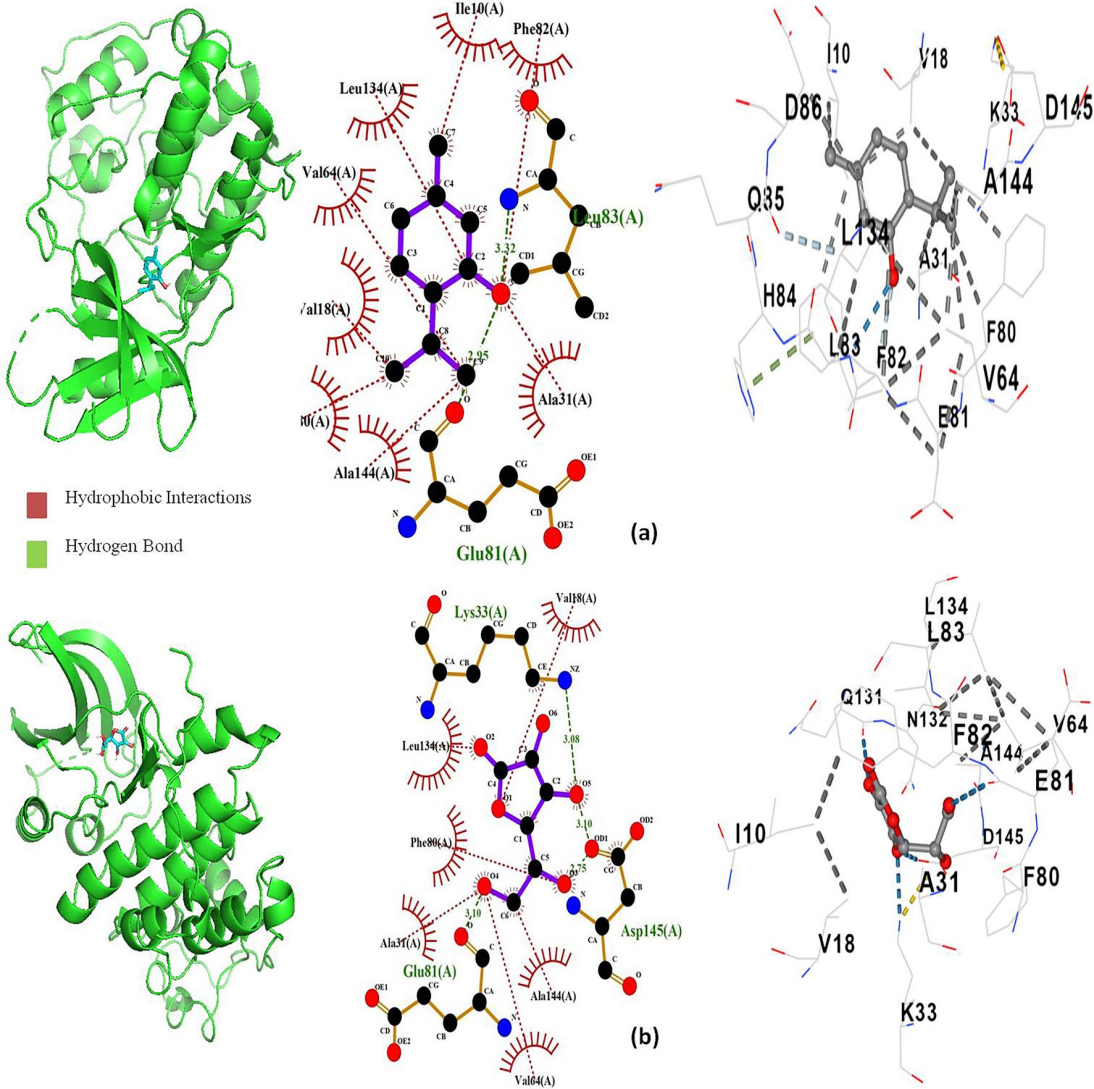
Interactions and docked 3D structures of (**a**) thymol with protein human cyclin-dependent kinase 2 complex 1HCK and (**b**) ascorbic acid with protein human cyclin-dependent kinase 2 complex 1HCK as control.

The molecular interactions between major volatile compounds like *p*-cymene, γ –terpinene, and thymol of *T. vulgaris* essential oil and the vital enzymes involved in biosynthesis and repair of cell walls, nucleic acids, and proteins in bacteria along with protein human cyclin-dependent kinase 2 complex and cyclooxygenase-2 are summarized in Table 5. All the abundant compounds were found to be most actively binding with D-alanine: D-alanine ligase (PDB ID: 2ZDQ) enzyme with the highest binding affinity of *p*-cymene (−7.9 kcal/mol) followed by γ –terpinene (−7.8 kcal/mol) and thymol (−7.7 kcal/mol). It was found that the DNA gyrase (PDB ID: 1KZN) depicted highest binding affinity towards thymol (−6.3 kcal/mol) with a hydrogen bond of bond length 3.01 Ǻ between hydroxyl group of thymol and ASP A:73 of 1KZN and also a hydrophobic interaction involving A chains of ALA47, ASN46, GLU50, ILE78, THR165, VAL43, VAL71, and VAL167, followed by *p*-cymene (−5.8 kcal/mol) and γ –terpinene (−4.6 kcal/mol). The difference in binding affinity of the three major compounds was very similar for the other bacterial protein receptors. A good binding affinity of cyclooxygenase-2 (PDB ID: 1CX2) enzyme protein with all abundant compounds was observed, where the binding affinity of γ-terpinene (−6.3 kcal/mol) and *p*-cymene (−6.3 kcal/mol) were the same, but thymol (−6.5 kcal/mol) showed slightly higher affinity due to presence of hydrogen bond of bond length 2.81 Ǻ between hydroxyl group of thymol and ARG A:376 of 1CX2. In addition, there were hydrophobic interactions involving A chains of ALA151, ALA378, ARG150, ASP125, ASN375, ILE124, PHE529, and THR149. The binding affinity of thymol towards the protein human cyclin-dependent kinase 2 complex (PDB ID: 1HCK) with a value of −6.4 kcal/mol was considered better than ascorbic acid (−5.0 kcal/mol). There were two hydrogen bonds each of bond length 2.95 Ǻ and 3.32 Ǻ present between the hydroxyl group of thymol with GLU A:81 and LEU A:83, respectively. There were also hydrophobic interactions involving A chains of ALA31, ALA144, ILE10, LEU134, PHE82, VAL18, VAL64, and PHE80. Since, thymol depicted the best docking scores for 1KZN (−6.3 kcal/mol), 1CX2 (−6.5 kcal/mol), and 1HCK (−6.4 kcal/mol), binding analysis was conducted to reveal the interactions between ligands and protein-binding sites (Figs. 2, 3).

**Table 5:** Binding free-energy values of major volatile compounds of *T. vulgaris* essential oil.

Ligand	Binding Free Energy ΔG (kcal/mol)								
	1JZQ*	1KZN	2VEG	2ZDQ	3RAE	3SRW	3UDI	1CX2	1HCK
γ -terpinene	−5.7	−4.6	−4.5	−7.8	−5.3	−5.6	−5.0	−6.3	−5.2
Ρ-cymene	−5.8	−5.8	−4.6	−7.9	−5.8	−5.6	−5.1	−6.3	−4.5
Thymol	−5.4	−6.3	−4.7	−7.7	−5.6	−5.7	−5.2	−6.5	−6.4
Ascorbic acid**	–	–	–	–	–	–	–	–	−5.0

*Protein PDB ID:1JZQ- isoleucyl-tRNA synthetase, 1KZN- DNA gyrase, 2VEG-dihydropteroate synthase, 2ZDQ-D-alanine:D-alanine ligase, 3RAE-topoisomerase 4, 3SRW-dihydrofolate reductase, 3UDI-penicillin-binding protein 1a, 1CX2- cyclooxygenase-2, and 1HCK- protein human cyclin-dependent kinase 2 complex. **Ascorbic acid: Used as a reference for antioxidant activity.

## Discussion

In vitro polyploidization using synthetic antimitotic agents can be an effective method to generate polyploid plants with enhanced biological traits. Still, it is not applicable every time as the result of gene duplication is unknown^46,47^. In the Lamiaceae family, polyploidization has drawn attention to crop improvement because of its potential to achieve higher secondary metabolites as well as increased essential oil content^34,35^. Therefore, it is important to analyze the effect of polyploidization on the biological activity of essential oils.

Previously, it has been reported that the average essential oil yield of *T. vulgaris* ranges between 0.3 to 1.2%^7^. In our study, we found that the essential oil yield of the diploid control was 0.85% whereas the polyploid genotype exhibited an increased amount of essential oil content (1.2%) which is an increase of 41.11% compared to the control diploid genotype. Similarly, the increased essential oil content in the induced polyploids of *T. vulgaris* has been reported previously^34,48^. The enhanced essential oil yield in polyploid plants has also been observed in other Lamiaceae family species such as *Mentha spicata*^35^ and *Tetradenia riparia*^49^. However, significantly lower essential oil content was observed in polyploid *Humulus lupulus* than in diploid as an effect of artificial polyploidization^50^. Although, there is not always an increase in essential oil quantity, however, our results indicated the potential to enhance essential oil quantity through synthetic polyploidization that can be used as an important tool for crop breeding.

Essential oil yield along with its phytochemical constituents can be affected by polyploidization^34^. GC–MS analysis revealed that the essential oil of both *T. vulgaris* genotypes consisted of three major components thymol, γ-terpinene, and *p*-cymene. When compared with the diploid control, thymol and γ-terpinene contents increased in tetraploid essential oil whereas *p*-cymene was found in higher amounts in the diploid control. These major compounds were reported to have antimicrobial, antioxidant, and anti-inflammatory activities^12,32,51,52^ and are also widely used in pharmaceutical and food industries. Similarly, Homaidan Shmeit et al.,^34^ and Navratilova et al.,^48^ have reported increased thymol and γ-terpinene contents in the polyploid *T. vulgaris* essential oil and decreased *p*-cymene content. However, a decrease in the amount of major compounds has been reported in some polyploid plants^53^. It can be assumed that the increased amount of these secondary metabolites in *T. vulgaris* polyploid essential oil is majorly responsible for its enhanced biological activities compared to the control diploid.

In this study, the antimicrobial activity on respiratory pathogens such as *H. influenzae*, *S. aureus*, *S. pneumoniae*, and *S. pyogenes* revealed that the induced tetraploid *T. vulgaris* essential oil has higher antibacterial activity in comparison to diploid control, although, both genotypes exhibited the best results in the liquid phase. Several works on antibacterial activity have been previously reported for diploid *T. vulgaris* essential oil that showed similar results to our findings^7,14^ but this is the first report on the antibacterial activity of induced tetraploid *T. vulgaris* essential oil. The antimicrobial activity of tetraploid essential oil against these tested microbes revealed that higher concentrations of abundant compounds may be majorly responsible for the increased antimicrobial activity in the tetraploid line as they have previously been well established for their antimicrobial activity^51,52^. The molecular interaction study revealed that thymol has a higher binding affinity towards DNA gyrase than other major compounds which is an essential target for antibacterial agents as It regulates DNA structure during transcription and replication by introducing breaks in both DNA strands, which is crucial for bacterial survival. Therefore, the higher amount of thymol content in polyploid essential oil probably contributes to its higher antibacterial activity. A similar increased antibacterial activity has been reported for tetraploid-induced *Mitracarpus hirtus* where the tetraploid line exhibited higher antibacterial activity against *S. aureus and B. subtilis*^54^.

The tetraploid essential oil showed an increased amount of DPPH radical scavenging activity which means it is a better hydrogen provider compared to the diploid control genotype. The compounds present in *T. vulgaris* essential oils contain conjugated carbon double bonds and hydroxyl groups that readily inhibit free radicals that lead to antioxidant effects^32^. Several works described significant results for the antioxidant activity of *T. vulgaris* essential oil^55–57^. However, this is the first reported study demonstrating the antioxidant activity of induced polyploid *T. vulgaris* essential oil. It can be assumed that chromosome doubling genetically influenced the secondary metabolite production which resulted in increased antioxidant activity in the tetraploid genotype. Previously, it was reported that the effect of colchicine-induced tetraploid *Citrus limon* exhibited higher antioxidant activity than the diploid genotype^58^. Another study reported that the radical scavenging activity of *Geranium macrorrhizum* was related to the plant ploidy level^59^. Also, the molecular docking study revealed that thymol has a high binding affinity towards the protein human cyclin-dependent kinase 2 complex more than the known antioxidant agent ascorbic acid. It can be expected that the effectiveness of polyploid *T. vulgaris* essential oil in scavenging the DPPH radical is probably due to the increased substantial content of monoterpenoids and monoterpenes that were previously identified as potential antioxidants^32,51^.

COX-1 and COX-2 are two cyclooxygenase isoforms. COX-2 is an inducible form that catalyzes the biosynthesis of pro-inflammatory prostanoids (actually, the role of both COX forms is much more complex). COX inhibitors are used to relieve acute and chronic pain and inflammation^60^. We observed slightly higher activity of essential oil isolated from tetraploid plants. The major compound of thyme essential oil thymol is known as a potent COX inhibitor^61^. Also, the docking study showed a higher binding efficacy of thymol with the cyclooxygenase-2 protein. Therefore, a slightly higher amount of thymol found in essential oil from tetraploid can contribute to its higher activity. However, it is possible that other compounds contained in essential oils could also influence the overall activity of essential oils. Polyploidization can result in the opposite effect as reported for *Gynostemma pentaphyllum* leaf extracts where diploid showed the strongest inhibitory effects on the expression of TNF-α, IL-6, and COX-2 mRNA^33^. However, our results indicate that polyploidization could be an effective strategy for obtaining plant products with enhanced bioactivity.

## Conclusions

In the current study, the characterization of valuable biological activities of oryzalin-induced polyploid *T. vulgaris* essential oil has been acquired for the first time. These findings also indicate the effectiveness of artificial polyploidization in *T. vulgaris.* The induced genotype exhibited a significant increase in essential oil yield with simultaneously higher concentrations of biologically active compounds such as thymol and γ-terpinene. The polyploid genotype exhibited enhanced antibacterial, antioxidant, and anti-inflammatory activities compared to the diploid genotype. Additionally, this study suggests that the induced genotype of *T. vulgaris* has improved traits that can be embraced for commercial use to obtain economic advantage, especially in the pharmaceutical and food industries due to the enhanced quantity and quality of essential oil. Synthetic polyploidization may perform a crucial role in the breeding of plants with high biological activities. However, further in vivo studies should be assessed to confirm their practical application in the above-mentioned industries.

### Supplementary Information

Supplementary Information.

## Data Availability

Data is provided within the manuscript or supplementary information files.
