# Supplementary material for: Synthetic polyploidization induces enhanced phytochemical profile and biological activities in Thymus vulgaris L. essential oil
Source: Sci Rep. 2024 Mar 7;14:5608. doi: 10.1038/s41598-024-56378-7 (PMC10920654; doi:10.1038/s41598-024-56378-7)
Supplement: Supplementary file 1 — Supplementary Information. [file 41598_2024_56378_MOESM1_ESM.docx]

**Original Article**

**Synthetic polyploidization induces enhanced phytochemical profile and biological activities in *Thymus vulgaris* L. essential oil.**

Neha Gupta^1, †^, Soham Bhattacharya^2, †^, Adrish Dutta^1^, Jan Tauchen^3^, Přemysl Landa^4^, Klára Urbanová^5^, Markéta Houdková^1^, Eloy Fernández-Cusimamani^1*^ and Olga Leuner^1^

^1^Department of Crop Sciences and Agroforestry, Faculty of Tropical AgriSciences, Czech University of Life Sciences Prague, Kamýcká 129, Suchdol, 165 00 Prague 6, Czech Republic.

^2^Department of Agroecology and Crop Production, Faculty of Agrobiology, Food and Natural Resources, Czech University of Life Sciences Prague, Kamýcká 129, Prague 6 – Suchdol, 165 00, Czech Republic.

^3^Department of Food Science, Faculty of Agrobiology, Food and Natural Resources, Czech University of Life Sciences, Prague, Czech Republic.

^4^Laboratory of Plant Biotechnologies, Institute of Experimental Botany of the Czech Academy of Sciences, Rozvojova 263, 165 02, Prague 6 – Lysolaje, Czech Republic

^5^Department of Sustainable Technologies, Faculty of Tropical AgriSciences, Czech University of Life Sciences Prague, Prague, Czech Republic.

**†These authors contributed equally to this work.**

***Address correspondence to**:

Eloy Fernández-Cusimamani

Department of Crop Sciences and Agroforestry, Faculty of Tropical AgriSciences, Czech University of Life Sciences Prague, Kamýcká 129, Suchdol, 165 00 Prague 6, Czech Republic.

Email: [eloy@ftz.czu.cz](mailto:eloy@ftz.czu.cz)


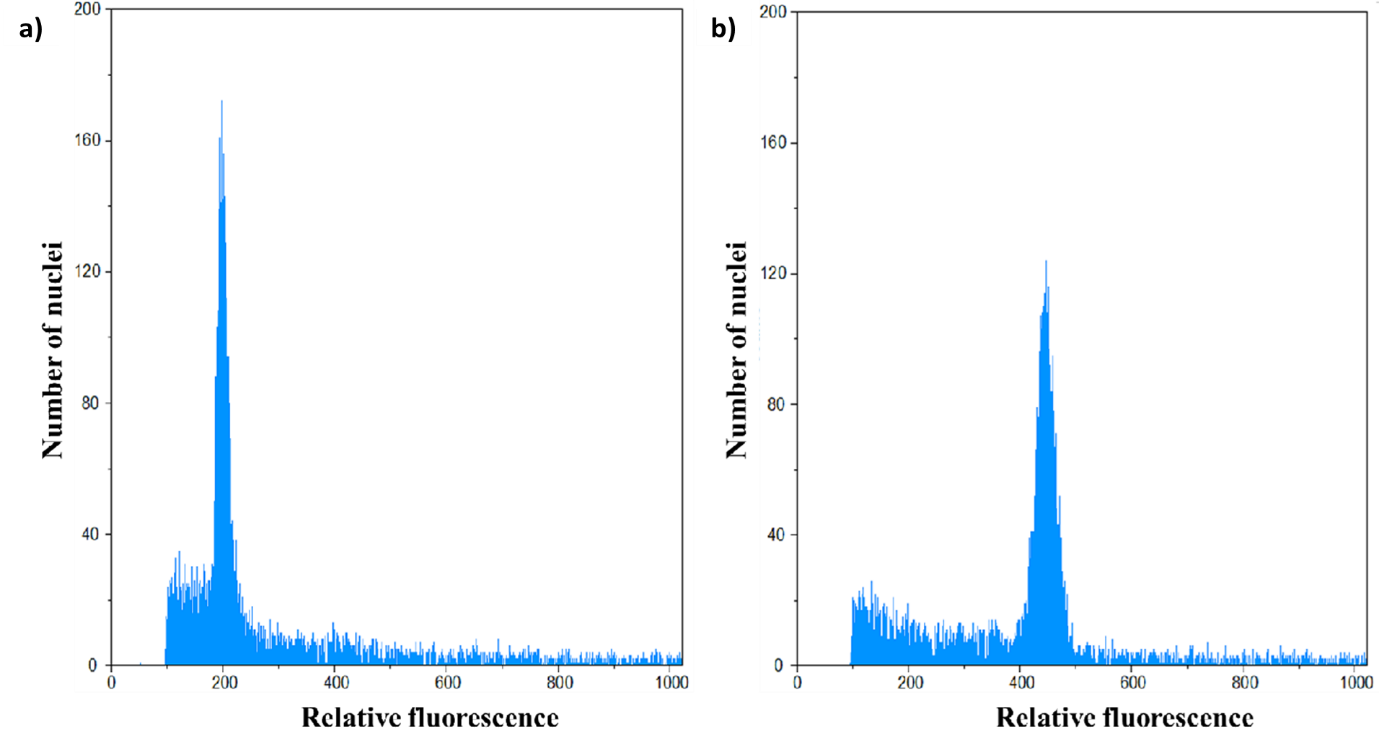
Supplementary Fig. S1.**:** Flowcytometric analysis of *T. vulgaris* (**a**) histogram of relative DNA content of control diploid and (**b**) tetraploid plant.


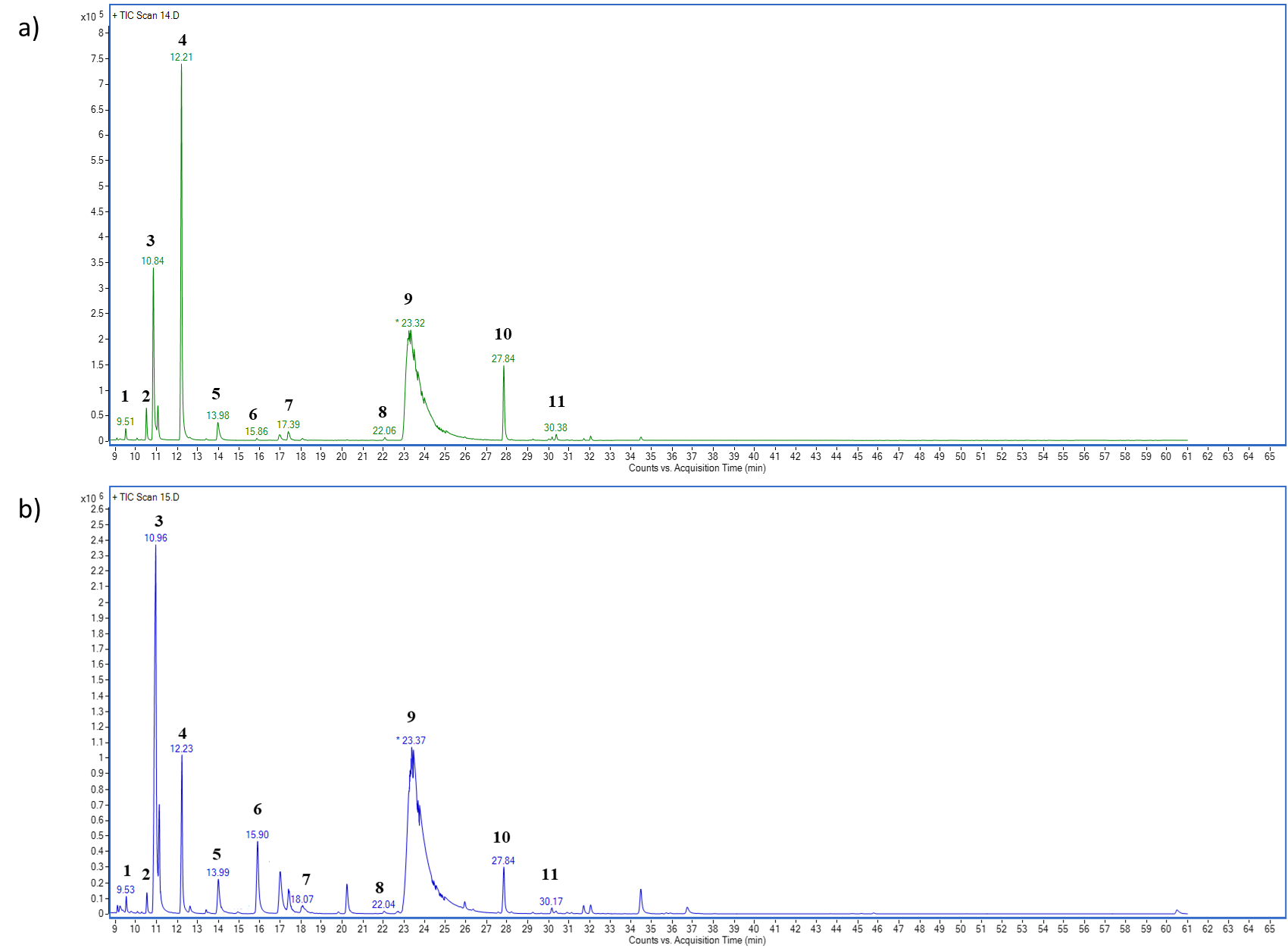


Supplementary Fig. S2.**:** GC-MS chromatograms of *Thymus vulgaris* L. essential oil obtained from a) tetraploid and b) diploid genotype. Peak number and compound names: 1. Mycrene, 2. 4-carene, 3. p-cymene, 4. γ-Terpinene, 5. Linalool, 6. d-camphor, 7. 4-Terpineol, 8. Borneol, 9. Thymol, 10. Caryophyllene, 11. D-Germacrene.
